# Memory instability as a gateway to generalization

**DOI:** 10.1371/journal.pbio.2004633

**Published:** 2018-03-19

**Authors:** Edwin M. Robertson

**Affiliations:** Institute of Neuroscience & Psychology, Centre for Cognitive Neuroimaging, University of Glasgow, Glasgow, United Kingdom

## Abstract

Our present frequently resembles our past. Patterns of actions and events repeat throughout our lives like a motif. Identifying and exploiting these patterns are fundamental to many behaviours, from creating grammar to the application of skill across diverse situations. Such generalization may be dependent upon memory instability. Following their formation, memories are unstable and able to interact with one another, allowing, at least in principle, common features to be extracted. Exploiting these common features creates generalized knowledge that can be applied across varied circumstances. Memory instability explains many of the biological and behavioural conditions necessary for generalization and offers predictions for how generalization is produced.

Our past experience can aid our current and future performance. For instance, being a skilled tennis player may help when it comes to playing other racquet sports such as squash. Encouraging the transfer of skill from one situation to another also lies at the heart of many brain training and rehabilitative strategies.

The ability to generalize from a specific example to a category or concept is not restricted to the world of actions. The location of a reward in a navigation task can be found more quickly in subsequent versions of the task even though key aspects of the task, including the location of the reward, change [1]. Similarly, generalization can occur across different objects, facts, or events to create categories. Generalization therefore plays a key role in a wide array of cognitive functions. Yet, despite its clear importance and adaptive value, how and when generalization occurs is poorly understood.

## Instability as opportunity

Generalization requires the identification of features common across experiences. For example, a common element of some navigation tasks is that a food pellet is never visible but is instead always buried within the sand. To discover the common feature requires a comparison and hence an interaction between memories for the different tasks. Interactions between memories can also lead to interference, after which a memory either is lost or becomes inaccessible [2]. In either case, knowledge is lost, and this loss, due to the formation of another interfering memory, is a disadvantage of a plastic and hence changeable network (for a review, please see [3]).

When memories are formed in quick succession, each is unstable and susceptible to interference [4,5]. The interference between memories may, in part, be due to overlapping or functionally connected patterns of activity [4]. Normally, discrete populations of neurons, or ensembles, are activated within the hippocampus when a memory is formed. These ensembles overlap when memories for different contexts are acquired within a few hours of one another [6,7]. However, when a substantial delay is introduced (i.e., >8 hours), there is no longer an overlap in the memory representations. As a consequence, the temporal profile of the overlap in memory representation matches the time interval that a memory is unstable following its formation [4]. In sum, the overlapping representation of memories may lead to interference, making them unstable for several hours after their formation.

**Fig 1:**
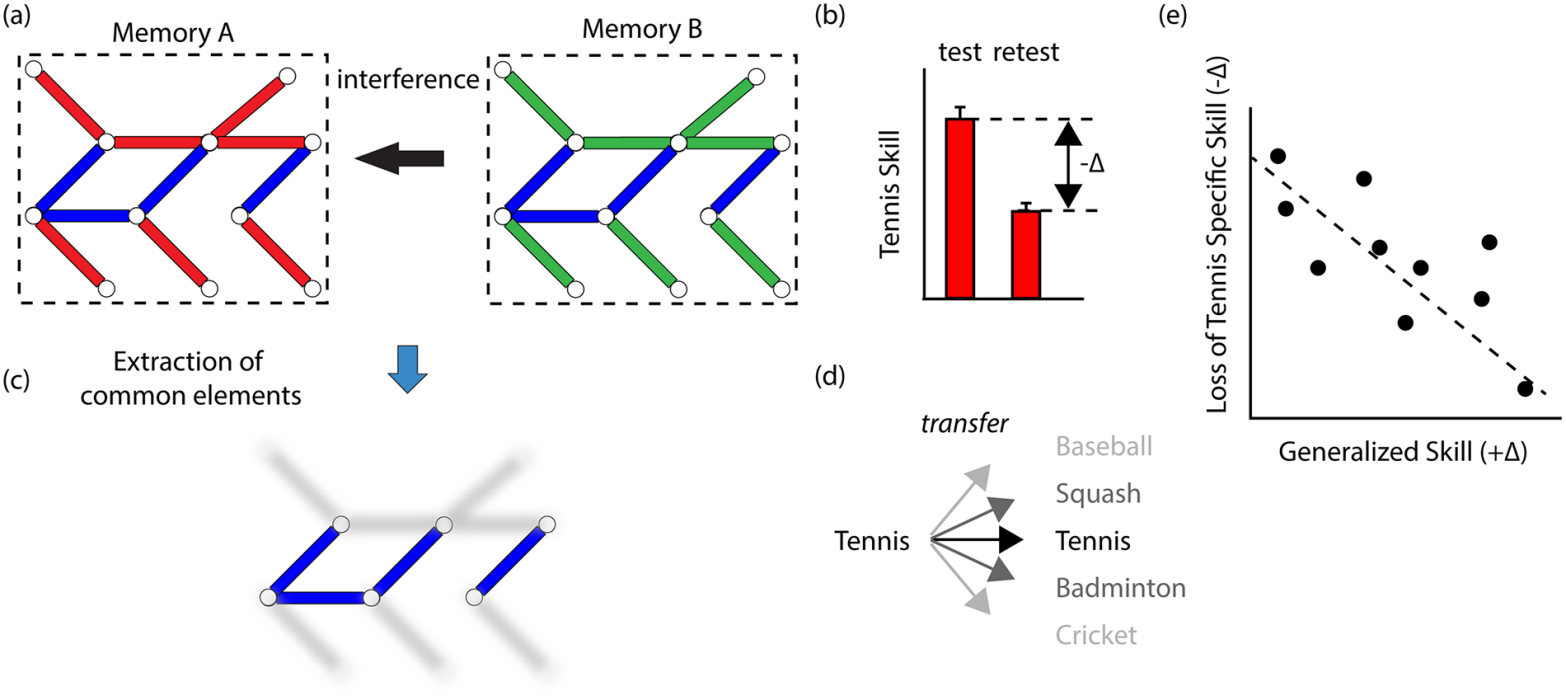
Memory instability as an explanation for the trade-off between detailed knowledge and generalization. Each memory representation has common (blue bars) and unique features (memory A, red bars; memory B; green bars). (A) When unstable memories interact and interfere with one another, it leads (B) to the loss of detailed information about an event or action. For example, learning tennis (memory A) and badminton (memory B) in quick succession might lead to loss of skills specific to tennis. (C) However, the interaction between memories may allow the identification and extraction of shared common features between memories (blue bars). (D) Exploiting those common features allows knowledge to be applied broadly across a range of related situations. For instance, the skill acquired playing tennis can be applied or transferred to other related racquet sports (dark grey; squash and badminton) and also perhaps even to other somewhat related sports (light grey; cricket and baseball). Instability provides an opportunity for interaction between memories, which can lead to their disruption and the loss of detailed knowledge, while simultaneously allowing shared features to be identified and exploited to allow generalization. As a consequence, (E) instability can explain the trade-off between detailed knowledge and generalization [5,8].

While instability leads to interference, and potentially to the loss of a memory, it also provides an opportunity for memories to interact with one another. Through these interactions, the statistical regularities shared between memories may activate common networks, strengthening them or at least distinguishing them in some way from those networks associated with the detailed differences between the memories. In this way, memories can, at least in principle, be compared, and shared features can be identified and potentially used to enable the creation of generalized knowledge that can be applied in different circumstances (Fig 1). Thus, whilst instability makes a memory vulnerable to forgetting, it is also linked to and perhaps even critical for the construction of generalized knowledge, which is central to so much of behaviour. This is not to suggest that instability is unique in supporting generalization (please see “Alternative mechanisms of generalization,” Box 1). Instead, what is explored here is memory instability as perhaps one gateway to generalization. Within this scenario, instability is adaptive, rather than purely detrimental, and offers a time window for the creation of generalizable knowledge (please see “Time window of generalization,” Box 2).

**Box 1. Alternative mechanisms of generalization**

Different mechanisms may be responsible for supporting generalization under different circumstances. Learning may enhance the plasticity of a circuit, which could improve the learning of any subsequent task [9]. For example, learning a sequence of movements can improve how quickly participants adapt their movements to a novel visual environment, as occurs in prism adaptation. As a mechanism for generalization, it is potentially broad because it does not require shared attributes or knowledge between tasks; however, it does require similar or at least partially overlapping circuits to be involved in learning the different tasks. Within this framework, learning primes neuroplastic mechanisms, supporting the transfer of performance to subsequent tasks.

Forgetting may also drive generalization [10]. Losing information that appears only in specific situations allows a memory to become less tied to a specific circumstance and thus able to be applied generally across a wide range of circumstances (forgetting model). By contrast, rather than losing irrelevant information and diminishing the specificity of a memory, it may be possible to identify relevant information, a pattern, or a feature that reoccurs across a range of situations, enhance knowledge for that feature, and thus increase the efficacy of the memory across a range of situations. Identifying these common features requires an interaction or communication between memories, which can occur when they are unstable (instability model; [5,7,11]). Thus, generalization might be achieved by losing knowledge for specific situations; equally, it may also be achieved by enhancing knowledge for features that are a motif across a family of tasks.

Identifying the information that has to be either strengthened or weakened is an important challenge for both of these models of generalization. This challenge is substantial because it is also potentially a dynamic challenge. Initially, a feature of a task could recur across a family of tasks, and thus, strengthening knowledge of this feature would aid generalization. Yet, later in another circumstance, this same feature might be an idiosyncrasy of one particular situation, and therefore, strengthening knowledge of this feature, rather than aiding generalization, would only serve to increase the specificity of a memory. Another feature that these models share is that they link generalization to the loss of detailed information. For one model, forgetting, the loss of knowledge, drives generalization by decreasing the specificity of a memory. Yet, in the other, it is a side effect. The interaction between unstable memories can lead to identifying common features between tasks, producing generalization, but it also leads to the disruption and loss of detailed knowledge [5]. Overall, the different models of forgetting and instability envisage generalization arising by weakening or strengthening different aspects of a memory (i.e., specifics versus recurring motifs, respectively). Both models share the common challenge of how to identify the information that needs to be strengthened or weakened, and both, either directly or indirectly, provide a link between generalization and the loss of detailed knowledge.

These are examples of how generalization may arise. Each mechanism is better suited to, and perhaps can only operate under, specific circumstances, and thus, it seems likely that at least in principle, these mechanisms could act together in a complementary fashion, with the strengths of one compensating for the weaknesses of another.

**Box 2. Time window of generalization**

Generalization develops over a diverse range of timescales, from hours to weeks to even years [5,7,8,12]. In some cases, generalization develops during those hours of instability following initial memory formation. For example, immediately after learning, performance can transfer from one sequence to a different type of sequence (action versus words) provided that the sequences share a common structure [5]. Equally, immediately following the formation of a memory, fear can transfer from one context to another neutral context [7]. In these examples, generalization develops within hours during a single episode of instability following memory formation.

On other occasions, generalization can take weeks and potentially years to develop [8,12]. Perhaps the features common across some tasks are so complex that they require multiple episodes of instability to be identified, which increases the time necessary for generalization to develop. Instability on multiple occasions may be possible because of memory replay during sleep or perhaps memory reactivation when other similar new memories are being encoded (for a short review, please see [13]). Even once a common feature between memories has been identified, other subsequent processes may be required for generalization to be expressed (for example, forgetting; see Box 1). In this scenario, instability is necessary to trigger the development of generalization but is not in itself sufficient. These processes might act together, in some cases, with multiple episodes of instability, triggering other complementary processes, which take time to develop, and subsequently support generalization.

## Common conditions

It is when memories are unstable that transfer is most prominent. For example, a recently acquired perceptual skill for detecting a stimulus at one location can easily generalize or transfer to other new locations [14]. Similarly, a newly acquired motor skill learnt with one hand can be easily transferred to the other hand (i.e., intermanual transfer; [15–18]). Yet, transfer is much reduced once a memory has been stabilized through consolidation [11]. Even following consolidation, it is only after a memory has once again become unstable that it can be modified and integrated with other memories, which is a prerequisite for subsequent generalization [19]. Together, these studies suggest that for many tasks, memory instability and transfer are found in similar circumstances. Yet, this is only circumstantial evidence. Few studies have measured memory instability and subsequent performance transfer together.

## Behaviour connects instability to generalization

Recent work has tested the link between instability and generalization [5]. Learning a sequence of actions improves subsequent learning of a sequence of words. Conversely, learning a sequence of words improves the subsequent learning of a sequence of actions. This reciprocal pattern of transfer between different types of knowledge occurs provided 2 conditions are satisfied. Firstly, the motor and word sequences must share a common abstract structure or grammar. What is transferred is the high-level or abstract relationship between elements rather than knowledge of the individual elements themselves (i.e., words versus actions). Secondly, the memory must be unstable for performance to transfer. The instability of the initial memory is correlated with subsequent transfer, suggesting that transfer is related to the instability of the memory. Yet, the relationship between instability and transfer goes beyond correlation. Stabilizing the initial memory, preventing it from being susceptible to interference, also prevented transfer to the subsequent memory task. Thus, transfer from one task to another was critically dependent upon memory instability.

## Modifying stability modifies transfer

A manipulation that modifies memory stability can also modify transfer. Prolonged practice stabilizes a memory [20]. Usually, a new skill memory is so unstable that it can be disrupted by subsequently learning another different skill [4]. Yet, when the amount of training is increased, the newly formed skill memory is no longer susceptible to interference from further learning. The increased training has stabilized the memory. The increased training also reduces subsequent skill transfer. For example, after a short period of initial training on a visual task, participants show substantial transfer to a novel visual task, whereas after prolonged training, there is limited transfer [14,21]. Similarly, the transfer of skill between hands is frequently greater when there is a short rather than a prolonged period of initial training [22]. Extended training can also lead to the formation of habits, which show limited transfer [23]. Yet, not all extended training leads to the formation of a habit. Critically, a habit also requires a reduction in the importance of a goal [24]. The common feature across habits and extended training is the duration of practice, and thus, it seems likely that this is responsible for impairing transfer. Together these studies suggest that a manipulation—specifically, prolonged practice—can both stabilize a memory and reduce transfer from a learnt to a novel task.

Prolonged practice leading to reduced transfer may seem counterintuitive. With prolonged practice comes increased proficiency, which might logically be expected to improve transfer. After all, the more knowledge is gained about one task, then the greater facility and hence perhaps the greater the potential to transfer performance to another related task. Yet, transfer does not seem to operate in this way. Prolonged practice appears to prevent rather than support transfer. Thus, transfer is not simply dictated by the accumulation of knowledge or performance. Instead, transfer occurs in the same circumstances as memory instability, is for at least one set of tasks critically dependent on instability, and can be prevented by prolonged practice, which stabilizes a memory. Together, these findings converge to suggest a link between memory instability and transfer.

## A common mechanism for stability and transfer

Prolonged practice leads not only to memory stabilization and impaired transfer; it also leads to neurochemical changes. One such change is an increase in GABA within the cortex. Potentially, this increase following prolonged practice may be responsible for stabilizing the memory and for reducing transfer.

Changes in GABA have been linked to changes in performance transfer. The concentration of GABA within the cortex can be modified using a noninvasive brain stimulation technique called direct current stimulation. In this technique, 2 electrodes (an anode and cathode) are placed on the scalp of a human participant, and a small current is passed between the electrodes (for a review, please see [25]). Placing the anode of a stimulation device over the motor cortex decreases GABA concentrations in this area [26]. Decreasing GABA in this way contralateral to the trained hand enhances subsequent transfer to the untrained hand [27]. Thus, GABA is linked to performance transfer: an increase in GABA, due to prolonged practice, impairs transfer, while a decrease in GABA, due to current stimulation, enhances transfer. Equally, an increase in GABA has been linked to an increase in memory stability [20,28]. Together, these studies reveal a mechanistic link between stability and transfer. Subsequent studies may further test the nature of this link by using pharmacological methods to specifically modify GABA. However, even with converging evidence to show that GABA mechanistically links stability with transfer, it should not be assumed that this link depends solely on GABA. Potentially, GABA is only one component of what is likely to be shown, in time, as a complex and diverse neurochemical mechanism linking memory stability to performance transfer.

## Circuits of stability

A link between memory stability and generalization is also present at the level of networks and brain circuits. One part of a network critical for generalization and for the interaction between unstable memories appears to be the prefrontal cortex.

Several studies have shown that the prefrontal cortex makes a critical contribution to generalization. For instance, lesions to the ventromedial prefrontal cortex in humans or disruption to prefrontal function with transcranial magnetic stimulation (TMS) prevents semantic generalization [29,30]. This is when participants learn a list of semantically related words and subsequently incorrectly identify another semantically related word as coming from the list [31]. These errors due to semantic generalization, frequently called false memories, are decreased when the function of the prefrontal cortex is impaired.

Other studies have shown that the prefrontal cortex makes a critical contribution to memory instability. Disrupting the function of the human prefrontal cortex, with TMS, prevents newly formed unstable memories from being susceptible to interference [32]. Similarly in rodents, a lesion to the frontal cortex also prevents the interaction between new unstable memories ([33]; for a review, please see [4]). Together, these studies suggest that the prefrontal cortex is responsible for creating interference between newly acquired memories [4].

The prefrontal cortex may support interference between memories by affecting their representation. Unstable newly formed memories have an overlapping representation within the hippocampus [6,7]. The prefrontal cortex exerts an influence upon the representation of motor skill memories in the primary motor cortex, and thus, at least in principle, it may have a similar role in influencing the representation of memories in the hippocampus [34,35]. Disruption to the prefrontal function could then transform the overlapping representation to a set of independent representations. Without an overlap, there may be no communication or interference between the memories, which is consistent with the work on rodents and humans [32,33]. Envisaging the overlapping representations as providing communication between memories would explain their vital contribution to transfer [7]. Information from one memory, for example, about the fear associated with one context has to be communicated to another memory for fear to transfer to a previously neutral context. Thus, instability and susceptibility to interference may be achieved by the prefrontal cortex creating overlapping memory representations, which are critical for transfer. This may explain the critical contribution of the prefrontal cortex to semantic generalization.

A similar mechanism may also allow generalization between old and recently formed memories. Previously formed memories are reactivated when related new information is being encoded into a memory. Reactivation of the memory is due to a dialogue between the medial prefrontal cortex and the hippocampus ([36–38]; for a review, please see [39]). When reactivated, an old memory becomes once again represented within the hippocampus, and it reverts to an unstable state, which allows it to be modified [40]; it can be then strengthened or integrated with a new memory [19,41]. The instability, modifiability, and capacity to be integrated with new memories suggest a communication between old and new memories, which may be achieved by the old reactivated memory sharing an overlapping representation with the new recently formed memory ([6]; for a review, please see [10]). Such communication between the memories may allow the identification of common elements, or motifs, which in turn supports the creation of generalizable knowledge. Once the common elements have been identified, the newly acquired memory may quickly cease to be represented within the hippocampus, instead becoming part of a cortical representation of the common properties shared across the old and new memory (i.e., part of a schema; [1]). Overall, the prefrontal cortex mediates the reactivation of an old memory, leading to it becoming once again unstable and able to have an overlapping representation with a new related memory, which provides perhaps the basis for communication between memories and generalization across them.

Prefrontal circuits are not unique in making a critical contribution to generalization. The circuit can alter depending upon the nature of the common characteristic or repeating regularity that is being generalized. For instance, a circuit that includes the ventromedial prefrontal cortex is critical for semantic generalization [29,30]. By contrast, when the common feature is no longer semantic but instead, for instance, spatial position, then another brain area, the angular gyrus, is critical for generalization across tasks [42,43]. Similarly, the circuit critical for a newly formed unstable memory alters depending upon the type of information learnt [44–47]. While the circuit dedicated to memory stability and generalization may vary, what does not vary perhaps is the overlapping relationship between stability and generalization.

## Instability: A trade-off between detail and generalization

Detailed knowledge can easily be lost when a newly formed memory is unstable and susceptible to disruption. For example, rather than recalling a complete list of 12 words, a person might only recall 10 words [5,32,48]. However, this loss of detailed knowledge can come with a benefit. There is a positive correlation between the loss of detailed knowledge and transfer of performance to a subsequent related task. For example, skill learnt in performing a sequence of actions is lost in direct proportion to the performance transferred to learning a sequence of words [5].

Similarly, knowledge of word sequence is lost in direct proportion to the performance transferred to an action sequence. This pattern of reciprocal transfer is observed when the sequence of words or actions share a common abstract structure. Converging with this behavioural work showing a trade-off between detailed knowledge and generalization is more recent functional imaging work (Fig 1, [8]).

Patterns of activation within the human hippocampus have also revealed a trade-off between detailed knowledge and generalization [8]. Maintaining a detailed knowledge of a learnt association between an object and a scene was measured as the match between the pattern of neural activation at the initial encoding and its subsequent retrieval. A tight match between the pattern of activation at encoding and retrieval indicated retention of detailed knowledge. Each of the 128 objects was uniquely paired with one of only 4 scenes. This allowed memories to be related to one another through a common shared scene. Similarity of activation between those memories with a shared common scene provided a measure of generalization. Using these analysis techniques provided measures of both detailed knowledge retention and generalization, which were negatively correlated to one another, revealing a trade-off between detailed knowledge retention and generalization. Overall, for both the behavioural and the functional imaging work, the greater the loss of detailed knowledge is, the greater the ability to generalize. This trade-off can be explained by memory instability being necessary for generalization.

Instability makes a memory susceptible to interference. The greater this instability is, the greater the interference, and the greater the loss of detailed knowledge. However, instability also increases the interaction between memories, potentially allowing the identification of shared common features, which can be exploited to allow transfer and generalization between different but related situations. Thus, instability may well explain the trade-off between the loss of detailed knowledge and generalization (Fig 1).

## Reward as a modifying factor of memory stability and generalization

After its formation, a memory is stabilized over several hours. Such offline processing can be affected by modifying factors, one of which appears to be reward. For example, providing a reward following learning for the retrieval of specific items enhances the subsequent recall of those items [49]. Similarly, rewarding the acquisition of a motor skill enhances the skill improvements that develop offline during consolidation [50].

The connectivity of circuits and what happens within those circuits following memory formation are both affected by reward. An increase in connectivity between the visual cortex and both the anterior hippocampus and the ventral tegmental area is associated with a reward [51]. There is also an increase in the frequency with which the pattern of neural activity present at memory formation is replayed offline during consolidation [52,53]. Together, these studies provide evidence that reward can affect offline memory processing during consolidation.

A wide range of memory changes occur during offline processing [54]. Stabilization is but one example of these changes. Other examples of offline processing, such as memory enhancement, are clearly affected by reward [50]; yet, what remains less clear is whether specifically memory stabilization is affected by reward. It is conceivable that a reward can affect memory stability. After all, reward leads to the release of dopamine, affecting synaptic plasticity mechanisms, which, at least in principle, may shorten the interval that a memory is unstable for [55,56]. This would suggest that reward might stabilize a memory. Increasing the stability of a memory with a reward may reduce the propensity to transfer learning between related tasks.

At first, the idea that reward will impair a behaviour, in this case the transfer of learning, may seem counterintuitive; however, it may have important adaptive benefits. Reward may be sculpting behaviour so that high performance is focused precisely on those tasks that yield a reward, and not upon related tasks that may not yield any reward. Overall, it seems highly likely that memory stability can be manipulated with reward; yet, what remains to be tested is whether this manipulation affects subsequent transfer between related tasks.

## Stressing memory

As is the case for a rewarding stimulus, a stressful or aversive stimulus can affect the offline processing of a memory. For example, in rodent studies, applying a foot shock immediately after memory formation can enhance subsequent consolidation [57]. Stress may act to stabilize a memory or at least reduce its susceptibility to interference by altering the connectivity of circuits so that memory processing becomes isolated or independent from other processing [58]. With this reduced vulnerability to interference, a memory is stabilized, and retention improved.

Whether these changes in the memory stability due to stress affect the development of generalizable knowledge is not currently clear. Some studies have suggested that stress increases generalization, others that it makes minimal difference, and others that it decreases generalization [59–61]. For some of these studies, stress did not increase knowledge retention, and thus, there is no evidence that stress affected consolidation or memory stability. When knowledge retention is increased, indicating enhanced memory stabilization, there is a decrease in generalization. The latter observations are consistent with the model proposed here, with stability favouring accurate detailed retention whilst instability favours generalization.

Memory instability is also implicated in the transfer of fear from one context to another. Fear paired within one context will transfer to another neutral context when, and only when, the acquisition of a memory for both contexts is separated by only a few hours (≈5 hours; [7]). There is no transfer when the interval between acquiring the memories is increased to 1 week. A similar time course is followed by memory instability, with a memory being unstable and susceptible to interference within the first hours of its formation and stable within a day (and certainly within 1 week) [4]. Instability and subsequent transfer are present over a similar time window. Thus, it is conceivable that the capacity to transfer fear between contexts is related to and, consistent with the current model, potentially relies upon memory instability.

Transfer is also linked to instability through how memories are represented. When acquired in quick succession, memories share a neural circuit, or ensemble. When the overlap between memory ensembles is decreased through aging, transfer of fear to a new context is impaired [7]. Conversely, when the overlap in aging rodents is rescued through experimental manipulation, the transfer of fear to a new context is once again possible. What this beautifully illustrates is that an overlapping representation is necessary for subsequent transfer. The overlapping representation, on the one hand, provides a means for different memories to interact, common features to be identified, and transfer to happen. Yet, on the other hand, it makes memories susceptible to interference and perhaps makes them unstable.

Overall, (A) reducing memory instability with aversive stimuli impairs transfer; (B) memory instability is transient, lasting for only a matter of hours, and it is only during this time window that transfer of fear from one context to another is possible; and (C) instability may be due to an overlapping memory representation, which is critical for transfer. Together, these results converge to suggest that memory instability may have an important role to play in transfer and the creation of generalizable knowledge (please see “Predictions,” Box 3).

**Box 3. Predictions**

Memory instability may provide one gateway to the development of generalizable knowledge. As a consequence, modifying memory stability through fear, reward, or even prolonged practice could modify subsequent transfer of performance across related tasks or situations. Reward and fear are both predicted to decrease transfer because both enhance consolidation and thus are assumed to increase memory stability. Evidence is accumulating that is consistent with this view; however, as yet, there is very little direct evidence, because no single study has modified memory stability using fear or reward, measured that change, and examined the subsequent effects, if any, in transfer. Such studies are not without challenges. For instance, the shift in processing from a goal-based to a habit-based strategy that fear promotes could couple performance more tightly to a particular context and thus impair transfer, regardless of changes in memory stability [57,62,63].

Another approach to testing the link between memory instability and transfer is to better understand the conditions necessary for transfer. Fear can only transfer from one context to another neutral context when the memories for each context are formed within a few hours of one another [7]. The transfer in these circumstances could be related to memory instability. Memories are unstable for a few hours after their formation, the same time interval during which transfer is possible. This suggests a link between memory instability and subsequent transfer. Certainly, the instability of a memory for a sequence is related to the subsequent transfer to a different type of sequence (actions versus words; [5]). However, these are very different types of transfer—the latter case requires identifying and using the common sequential attributes to transfer performance, whereas in the former, there is no common element; instead, fear is being misattributed to a neutral context. Despite these differences, both may be dependent upon memory instability to enable transfer; alternatively, these differences may translate onto different mechanisms (see Box 1).

Transfer is also dependent upon the nature of the memory representation. When learnt within hours of one another, memories have overlapping representations [6,7]. Elegant work has shown that these overlapping representations are critical for transfer [7]. Manipulations that modify transfer would therefore be predicted to alter this memory representation and stability. For example, prolonged practice may diminish transfer and increase memory stability by promoting the creation of nonoverlapping or independent representations. The rise in GABA during prolonged practice may be responsible for diminishing the excitability within a shared overlapping representation and split it into independent representations [20]. Increasing excitability between independent representations can rescue the capacity to transfer fear from one context to another [7]. Manipulating memory representations may provide a way to test for a mechanistic link between instability and transfer.

## Brain state

Sleep has been linked to supporting generalization (for a review, please see [64]). During sleep, memories continue to be processed, are enhanced, and are reorganized [54]. The reorganization of past events potentially allows hidden patterns to be uncovered.

For instance, infants demonstrate knowledge for an artificial grammar of nonsense letter strings only after sleep during a nap. This is achieved by identifying repeating patterns—in this example, the grammatical structure common to letter sequences. Specifically, the first syllable (“PEL”) predicts the final syllable, and as a consequence, both “PELwadimRUD” and “PELchilaRUD” are valid grammatical structures for these nonsense letter strings [65,66].

Sleep has also been implicated in transitive inference when the high-order structure of the relationship between arbitrary symbols (such as fractal patterns) is uncovered based solely upon exposure to low-order relationships [67]. For example, when participants are exposed to simple pairings such as A > B, B > C, and C > D, the appropriate inference from exposure to these is that A > D, which is enhanced over sleep. Thus, sleep provides an environment that promotes the extraction of rules, the identification of repeating abstract patterns, and generalization across tasks.

Generalization during sleep may be linked to memory instability. Memories are reactivated during sleep, which may cause the memory to become unstable. The pattern of neural activity present during memory formation can be found again during sleep. For example, the neuronal firing patterns during a motor learning task are replayed again in the motor cortex of a rodent during sleep, and this replay is correlated with the subsequent sleep-dependent performance improvements [13,68]. These reactivations may lead to the memory becoming unstable [69]. When retrieved during wakefulness, a memory is rendered unstable, vulnerable to interference, just as the memory had been soon after its initial formation. Similarly, the reactivation of a memory during sleep may also make it unstable.

Elegant work has demonstrated that memories can be artificially reactivated during sleep [70,71]. For instance, a memory formed while a sensory cue, such as an odour, is presented can be reactivated when that same sensory cue is represented during sleep [70]. The same pattern of functional activation found during learning is found again when the sensory cue is represented during sleep.

Yet, when a specific memory is reactivated during sleep, it remains invulnerable to interference [72]. Interference from further learning is only one measure of memory instability, and a memory may be unstable, despite not being susceptible to interference. Changes in brain state during sleep may make a memory, even an unstable memory, invulnerable to interference.

During large parts of sleep, the effective functional connectivity of the human brain is markedly reduced. For instance, the waveform evoked by applying TMS to the motor cortex travels a substantially shorter distance when applied during slow-wave sleep than during wakefulness [73,74]. Along with this decrease in functional connectivity, there is a change in brain organization. Specifically, the brain becomes more modular. Functionally connected circuits remain, but these circuits are smaller and more constrained, lacking the widespread connections present during wakefulness [75].

Thus, memories may well become unstable over sleep but remain protected from interference because of the poor functional connection amongst brain areas. Unstable memories are constrained within functionally discrete, independent circuits, and therefore, interference between memories is minimized. Thus, sleep may provide an ideal environment for memories to become unstable because they are protected from interference. However, this environment may also restrict the generalization that is possible during sleep.

With connectivity limited during sleep, generalization may only occur between those memories represented within restricted circuits. This may mean that generalization can only occur across memories with certain properties such as having the same or similar content. By contrast, generalization can occur between memories with different content during wakefulness (i.e., between actions and words; [5]). Yet, transient restorations in long-range connectivity associated, for example, with sleep spindles may be sufficient to allow communication across brain areas to support generalization across diverse memories [76–78]. Alternatively, interludes of rapid eye movement (REM) sleep may be sufficient to restore connectivity when, or if, coordinated with episodes of memory reactivation that predominately occur when connectivity is reduced during slow-wave sleep [64]. Sleep may restrict the damaging effects of interference upon memories by having generally limited connectivity whilst simultaneously having brief restorations in connectivity to allow communication and potentially generalization across memories.

## Conclusions and beyond

Broadly, there appear to be at least 2 contrasting perspectives on memory instability, which are unlikely to be mutually exclusive. One perspective sees instability as arising from the unique requirements of biology. For instance, it takes time to synthesize the protein necessary to stabilize a memory, and thus, an interval of instability follows. From this perspective, memory instability is simply the inevitable consequence of having algorithmic processes implemented within a biological substrate. Implement those same processes within a different substrate, such as silicon, and memory instability may well vanish without any loss of memory function. In an alternative perspective, memory instability—and potentially the offline processing of memories more broadly—may make an indispensable contribution to the algorithms necessary for memory function. Instability may provide an opportunity for a particular form of computation or algorithm that is critical for memory function.

Instability may be critical to uncovering patterns common across different memories. It provides an opportunity for a comparison between different memories, allowing common features to be identified, extracted, and exploited. Once stabilized, a memory becomes invulnerable to interference; yet, it may also lose its ability to interact with other memories, and thus, features common to the memories can no longer be identified. Consistent with this idea, the transfer of performance from one task to another is most prominent in those circumstances that favour memory instability [11,14–18,20]. Yet, this is more than simply a circumstantial link. The transfer of performance across related sequential tasks is correlated with memory instability [5]. Stabilizing those memories, either through subtle changes to the tasks or inserting a time interval to allow consolidation to take place, prevents transfer [5]. Similarly, prolonged practice stabilizes perceptual memories and is associated with decreased transfer [14,20,21]. Conversely, reversing those neurochemical changes associated with memory stabilization enhances transfer [20,26–28]. Instability then may be critical for generalization. Instability also explains the trade-off between the loss of detailed knowledge and the creation of generalizable knowledge found in behavioural and functional imaging work (Fig 1; [5,8]). This relationship between instability and generalization may remain even in other brain states. Sleep has been widely associated with promoting generalization, and it is during sleep that the patterns of neural activity present during memory formation are replayed. Memories are rendered unstable through replay yet are protected from interference because of the changes in brain organization and connectivity that take place during sleep. Overall, from across a diverse array of studies, a consistent link emerges, connecting memory instability to generalization.

## Abbreviations

REM: rapid eye movement
TMS: transcranial magnetic stimulation
